# Targeting Molecular Inflammatory Pathways in Granuloma as Host-Directed Therapies for Tuberculosis

**DOI:** 10.3389/fimmu.2021.733853

**Published:** 2021-10-20

**Authors:** Reto Guler, Mumin Ozturk, Solima Sabeel, Bongani Motaung, Suraj P. Parihar, Friedrich Thienemann, Frank Brombacher

**Affiliations:** ^1^ International Centre for Genetic Engineering and Biotechnology, Cape Town Component, Cape Town, South Africa; ^2^ Department of Pathology, University of Cape Town, Institute of Infectious Diseases and Molecular Medicine (IDM), Division of Immunology and South African Medical Research Council (SAMRC) Immunology of Infectious Diseases, Faculty of Health Sciences, University of Cape Town, Cape Town, South Africa; ^3^ Wellcome Centre for Infectious Diseases Research in Africa (CIDRI-Africa), Institute of Infectious Disease and Molecular Medicine (IDM), Faculty of Health Sciences, University of Cape Town, Cape Town, South Africa; ^4^ General Medicine & Global Health, Cape Heart Institute, Faculty of Health Sciences, University of Cape Town, Cape Town, South Africa; ^5^ Department of Medicine, Faculty of Health Sciences, University of Cape Town, Cape Town, South Africa; ^6^ Department of Internal Medicine, University Hospital Zurich, University of Zurich, Zurich, Switzerland

**Keywords:** tuberculosis, host-directed drug therapy, lung pathology, granuloma, inflammation

## Abstract

Globally, more than 10 million people developed active tuberculosis (TB), with 1.4 million deaths in 2020. In addition, the emergence of drug-resistant strains in many regions of the world threatens national TB control programs. This requires an understanding of host-pathogen interactions and finding novel treatments including host-directed therapies (HDTs) is of utter importance to tackle the TB epidemic. *Mycobacterium tuberculosis* (Mtb), the causative agent for TB, mainly infects the lungs causing inflammatory processes leading to immune activation and the development and formation of granulomas. During TB disease progression, the mononuclear inflammatory cell infiltrates which form the central structure of granulomas undergo cellular changes to form epithelioid cells, multinucleated giant cells and foamy macrophages. Granulomas further contain neutrophils, NK cells, dendritic cells and an outer layer composed of T and B lymphocytes and fibroblasts. This complex granulomatous host response can be modulated by Mtb to induce pathological changes damaging host lung tissues ultimately benefiting the persistence and survival of Mtb within host macrophages. The development of cavities is likely to enhance inter-host transmission and caseum could facilitate the dissemination of Mtb to other organs inducing disease progression. This review explores host targets and molecular pathways in the inflammatory granuloma host immune response that may be beneficial as target candidates for HDTs against TB.

## Introduction

*Mycobacterium tuberculosis* (Mtb) is the causative agent of tuberculosis (TB). After COVID-19, TB remains the second leading cause of death from a single infectious agent with an estimated 1.4 million global deaths in 2020 (1). One of the major bottlenecks for global TB control is the emergence of multidrug-resistant and extensively drug-resistant TB. In 2019, half a million people developed rifampicin-resistant TB. People with TB disease are subjected to long treatment durations and drug side effects often lead to poor adherence, thus increasing the risk of developing drug-resistance. New treatment approaches are urgently needed to control TB. Mtb exploits and hijacks factors in the host to establish survival and persistence. Targeting host factors has emerged as novel TB treatment approaches termed host-directed drug therapies (HDTs). Developing novel HDTs for TB by targeting the host rather than the pathogen will circumvent conventional antibiotic resistance.

**Figure 1:**
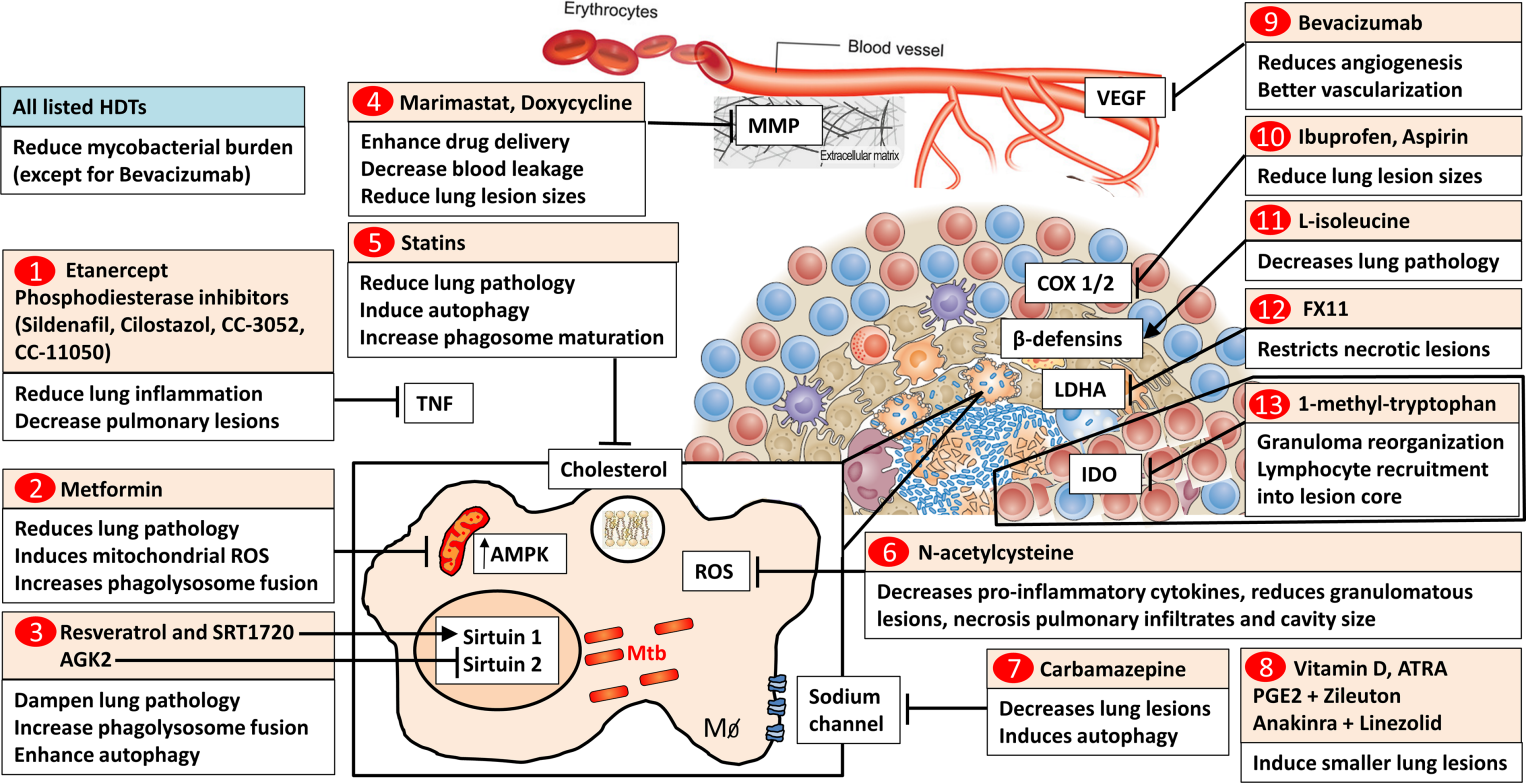
Host-directed drugs targeting molecular pathways within lung granulomas. 1) TNF blockers such as soluble TNF receptor 2 fusion protein, etanercept and phosphodiesterase inhibitors (Sildenafil, Cilostazol, CC-3052, and CC-11050) reduce lung inflammation and pulmonary pathology. 2) The antidiabetic drug metformin inhibits mitochondrial respiratory-chain complex 1 and increases AMPK levels. Metformin reduces lung pathology and in Mtb-infected macrophages induces mitochondrial ROS and increases phagolysosome fusion. 3) Histone deacetylase Sirtuin 1 activators (Resveratrol, SRT1720) and Sirtuin 2 inhibitor (AGK2) dampen lung pathology, increase phagolysosome fusion and autophagy. 4) Broad-spectrum metalloproteinase (MMP) inhibitor, Marimastat, enhances anti-tubercular drug delivery and reduces blood vessel leakage. Doxycycline reduces lung lesion sizes. 5) Statins, cholesterol-lowering drugs, reduce lung pathology. In macrophages, statins induce autophagy and increase phagosome maturation. 6) The antioxidant N-acetylcysteine (NAC) decreases pro-inflammatory cytokines, reduces tuberculous granuloma lesions, necrosis, pulmonary infiltrates, and cavity size. 7) Carbamazepine, a sodium channel blocker, decreases lung lesions and induces autophagy in macrophages. 8) Vitamin D and Vitamin A metabolite (all-trans retinoic acid, ATRA), PGE2 and Zileuton, Anakinra and Linezolid induce smaller lung lesions. 9) VEGF blocker (Bevacizumab) reduces angiogenesis and induces functionally better vascularized granulomas. 10) Nonsteroidal anti-inflammatory drugs (Ibuprofen and Aspirin) block cyclooxygenases and reduce lung lesion sizes. 11) The amino acid _L_-isoleucine decreases pulmonary pathology through the induction of β-defensins. 12) Lactate dehydrogenase A inhibitor (FX11) restricts necrotic lung lesions. 13) The IDO inhibitor, 1-methyl-tryptophan, results in the reorganization of granuloma architecture increasing lymphocyte recruitment into the lesion core. 1-13) All listed HDT candidates reduce mycobacterial burden except for bevacizumab. TNF, Tumor Necrosis Factor; AMPK, AMP-activated protein kinase; Sirtuin, Silent mating type information regulation 2 homolog; ROS, Reactive Oxygen Species; MMP, metalloproteinases; VEGF, Vascular endothelial growth factor; COX, cyclooxygenases; LDHA, Lactate dehydrogenase A; FX11, 7-Benzyl-2;3-dihydroxy-6-methyl-4-propyl-naphthalene-1-carboxylic; IDO, indoleamine 2;3-dioxygenase; ATRA, All-trans retinoic acid; PGE2, Prostaglandin E2. Image of the granuloma structure adapted from reference (3), Nature Publishing Group. Image of the blood vessel structure adapted from reference (8), Wiley Publishing Group (8).

Mtb is primarily transmitted by the respiratory route and the disease mainly affects the lungs. Once the TB bacillus infects the lungs it induces persistent inflammation, a main ingredient in the disease pathogenesis. In the lungs, Mtb infection leads to host inflammatory responses which enhances the destruction of the lung tissues causing pulmonary lesions and pathogen replication sites. Thus, the identification of pulmonary host targets for HDTs will aim to limit immune pathological lung destruction and increase host immune responses to control Mtb proliferation and persistence. The formation of granulomas in the lungs is a prominent hallmark during TB disease progression and is originally believed to provide a host-protective cellular structure to contain Mtb infection. The classical structure of the granuloma contains a necrotic center with a surrounding lymphocytic cell layer to control the spread of TB bacilli (2). Within granulomas, mycobacteria are residing intracellularly in monocyte-derived macrophages, foamy macrophages, epithelioid cells and multinucleated giant cells (3, 4). These cells present antigens to T cells for the secretion of various cytokines and chemokines inducing cellular recruitment or killing of intracellular mycobacteria *via* IFN-γ mediated release of reactive oxygen intermediates or reactive nitrogen intermediates (5). Additionally, cytotoxic T lymphocytes (CTLs) can kill Mtb-infected cells *via* granzymes, and granulysin or induce apoptosis (6). However, more recent studies elucidated that granuloma formation is regarded as a central point of immunopathogenesis during Mtb infection (4). Mtb’s ability to persist in granulomas for a long time raises the possibility that TB bacilli can utilize the granuloma as a safe shelter (7). Targeting host factors that can influence the tuberculous granuloma could therefore become an effective HDT approach for TB. In this review, we discuss host-specific Food and Drug Administration (FDA)-approved drugs and inhibitors (**Figure 1** and **Table 1**) that possess immunomodulatory activities in the lungs. We focused on HDTs that were evaluated in preclinical animal models or *ex vivo* granuloma models to reduce granulomatous lesions and ameliorate pulmonary inflammatory tissue pathology.

**Table 1:** List of host specific FDA-approved drugs and inhibitors that were demonstrated in pre-clinical animal studies to have immunomodulatory activities decreasing the mycobacterial burden and dampening lung pathology (fold changes were extracted by using GetData Graph Digitizer).

HDT class	Drug	Brand name	Target mechanism	FDA approved disease target	Half-life (hours)	Mtb CFU reduction in animals	Lung pathology reduction in animals	Clinical trial	REF
MMP inhibitors	Marimastat	No brand name	Broad-spectrum MMP inhibitor	Not approved	8-10	1.1 log in mouse lungs INH *vs* INH + Marimastat	2.3 fold reduced consolidated lung regions	Not started	(9)
MMP inhibitors	Doxycycline	Vibramycin	Multiple MMP inhibitor	Bacterial infection	16	1.6 log in guinea pig lungs	reduced area of granulomatous involvement	Phase 2 NCT02774993	(10)
Antioxidants	N-acetylcysteine	No brand name	Antioxidants	Paracetamol overdose	5.6	1 log in guinea pig spleen	3 fold necrosis score reduction in lungs	Phase 2 NCT00564642	(11)
TNF blockers	Etanercept	Enbrel	Soluble TNFR fusion protein	Rheumatoid arthritis	101	3.5-7.5 fold in Kramnik mouse lungs RHZ *vs* RHZ+ Etanercept	2.5 fold reduced lung involvement RHZ *vs* RHZ+ Etanercept	Not started	(12)
Phosphodiesterase inhibitors	Sildenafil	Viagra Revatio	PDE-5 inhibitor	Erectile dysfunction Pulmonary arterial hypertension	3-5	RHZ + Sildenafil + Cilostazol decreased time to lung sterilization by 1 month in mice	1.4 fold fewer lung lesions	Not started	(13)
Phosphodiesterase inhibitors	Cilostazol	Pletal	PDE-3 inhibitor	Intermittent claudication	11-13	1.9 log in mouse lungs	1.8 fold fewer lung lesions RHZ *vs* RHZ + Cilostazol	Not started	(13)
Phosphodiesterase inhibitors	CC-3052	No brand name	PDE-4 inhibitor	Not approved	Not known	0.8 log in rabbit lungs INH *vs* INH + CC-3052	2.7 fold fewer lung lesions INH *vs* INH + CC-3052	Not started	(14)
Phosphodiesterase inhibitors	CC-11050	No brand name	PDE-4 inhibitor	Not approved	Not known	1 log in rabbit lungs INH *vs* INH + CC-11050	2 fold fewer subpleural lesions INH *vs* INH + CC-11050	Phase 2 NCT02968927	(15)
Histone deacetylases	Resveratrol	No brand name	Sirtuin-1 activator	Not approved	2-4	0.8 log in mouse lungs	1.1 fold reduced involvement of lung parenchyma	Not started	(16)
Histone deacetylases	SRT1720	No brand name	Sirtuin-1 activator	Not approved	Not known	0.5 log in mouse lungs	1.2 fold reduced involvement of lung parenchyma	Not started	(16)
Histone deacetylases	AGK2	No brand name	Sirtuin-2 inhibitor	Not approved	Not known	0.8 log in mouse lungs	granuloma score: 5 fold reduced	Not started	(17)
Autophagy inducer	Carbamazepine	Tegretol	Sodium channel blocker	Epilepsy	27-37	1.7 log in mouse lungs	reduced lung lesions scores by 1.4 fold	Not started	(18)
Nonsteroidal anti-inflammatory drugs	Ibuprofen	Brufen	COX-1 COX-2 inhibitors	Painkiller	1-2	1.2 log in Kramnik mouse lungs	3.8 fold reduction of total lung affected area	Phase 2 NCT02781909	(19)
Nonsteroidal anti-inflammatory drugs	Aspirin	Bayer Aspirin	COX-1 COX-2 inhibitors	Fever, pain, inflammation	0.25	1 log in mouse lungs	decreased lung pathology by 3 fold	Phase 2 NCT02237365	(20)
Anti-diabetic	Metformin	Glucophage	Mitochondrial ROS AMPK	Type 2 diabetes	4-8	0.4 log in mouse lungs	reduced pathological lung area by 2.6 fold	Phase 2 NCT04930744	(21)
Vitamins	Vitamin D	Cholecalciferol	Nutritional supplementation	Nutritional supplements	360	0.6 log in mouse lungs PZA *vs* PZA + Calcitriol	1.4 fold reduction in lung lesions PZA *vs* PZA + Calcitriol	Phase 3 NCT00419068	(22)
Vitamin A metabolite	All-trans retinoic acid (ATRA)	Altreno	Binds to nuclear receptors, retinoid x receptor and RAR-a	Cancer	0.5-2	1.9 log in mouse lungs 0.5 log in rat lungs	reduced lung lesions in rats but not quantified	Not started	(23, 24)
Tryptophan catabolism	1-methyl-tryptophan	NLG802	IDO inhibitor	Not approved	10.5	1.5 log in mouse lungs	2 fold reduction in lung involvement	Not started	(25)
Amino acid	L-isoleucine	Aminosyn II 7%	Defensin activator	Nutritional supplement	3.5	3.5 fold in mouse lungs	decreased pneumonic area by 3.4 fold	Not started	(26)
Lactate metabolism	FX11	No brand name	Lactate dehydrogenase A inhibitor	Not approved	Not known	0.7 log in mouse lungs	reduced lung necrotic lesions by 1.9 fold in NOS2-/- mice	Not started	(27)
Prostaglandins	Prostaglandin E2 (PGE2)	Cervidil	Regulates cellular responses to PGE2	Gestational Trophoblastic Disease	0.06	PGE2 + Zileuton 0.8 log in IL-1aIL-1b-/- and 2.1 log in pICLC-treated mouse bronchoalveolar lavage fluid	PGE2 + Zileuton 1.6 fold reduced lung inflamed areas in IL1R1 deficient mice and 1.4 fold in pICLC-treated mice	Not started	(28)
Iron binding	Zileuton	Zyflo	Catalyst in leukotriene biosynthesis	Asthma	2.5			Not started	(28)
Transmembrane signaling receptor	Anakinra	Kineret	Regulates cellular responses to IL-1a, IL-1b and IL-1RN	Idiopathic Recurrent Pericarditis	4-6	Anakira + Linezolid 0.4 log in lung macaques	Anakira + Linezolid significant reduction in total lung FDG activity in macaques throughout treatment	Observational NCT04015713	(29)
Antibiotics	Linezolid	Zyvox	Binds prokaryotic 23S rRNA	Community Acquired Pneumonia (CAP)	5-7			NCT02778828	(29)
Statins	Simvastatin	Zocor	HMG-CoA reductase inhibitors to lower cholesterol	Cardiovascular diseases	4.6	2 fold in mouse lungs	1.2 fold reduced lung lesions	Not started	(30)
Statins	Rosuvastatin	Crestor	HMG-CoA reductase inhibitors to lower cholesterol	Cardiovascular diseases	14.2	0.5 log in mouse lungs	1.3 fold reduced lung lesions	Not started	(30)
Statins	Pravastatin	Pravachol	HMG-CoA reductase inhibitors to lower cholesterol	Cardiovascular diseases	2.2	0.9 log in Kramnik mouse lungs RHZE *vs* RHZE + Pravastatin	3.5 fold in lung involvement RHZE *vs* RHZE + Pravastatin	Phase 2 NCT03882177	(31)

## Granuloma in Latent and Active TB Patients

About 10% of individuals infected with Mtb develop active TB, while 90% remain lately infected and do not progress to active TB disease. Individuals with latent tuberculosis infection (LTBI) are able to contain the mycobacterial spread within distinct lung lesions where the TB bacilli can persist in a dormant state but can reactivate upon immune suppression (5, 32). The physical location and the physiological state of the persister bacilli still remain poorly understood (33, 34). Tuberculoma lesions in patients with latent infection are surrounded by highly vascularized tissues with high proliferative activity indicating host immune activation to contain the mycobacterial spread (5, 32). In contrast, the cavitary tuberculous lung lesions seen in active TB patients display low vascularization and low proliferative activity within the periphery of the lesions. Importantly, the wall structure in LTBI reveals a highly organized structure while the wall composition of cavities from active TB patients is diffuse and disorganized (5, 32). Despite individual heterogeneity, active TB patients display distinct pulmonary pathological characteristics that include hypoxia, caseous necrosis, and liquefied cavities containing large numbers of replicating bacilli (35). Understanding the dynamics of LTBI granuloma and progression into active TB granulomas can offer a plethora of HDTs.

## Granuloma Models

Various animal models have been used to study granulomas during infection with Mtb (35). Mouse models have been extensively used for TB drug efficacy studies and have shown similar potency between intravenous, low or high-dose aerosol infection models (36). However, mouse strains lack lung cavitation and caseating granulomas as observed in human granulomas. The mouse granuloma is non-hypoxic, lacks central necrosis and is loosely composed of macrophages, T lymphocytes and sporadic fibrous connective tissue (37). C3HeB/FeJ Kramnik mice develop highly organized lung necrotic lesions with evidence of hypoxia following infection with Mtb (38). Kramnik mice develop central liquefactive necrosis containing extracellular mycobacteria and their lung lesions are encapsulated with a fibrous layer. Bearing a resemblance to human lung lesions, the Kramnik mouse model is an important tool to evaluate the efficacy of anti-TB drugs, mainly for mycobacteria that can persist within necrotic lesions (39). Granulomas in zebrafish resemble key structural features of granulomas in humans, including a necrotic caseous core and an outer layer of epithelial cells that may restrict drug delivery (40, 41). Infection with *Mycobacterium marinum* of adult zebrafish causes caseating granulomatous TB (40) allowing for real-time visualization of granuloma formation in the optically transparent zebrafish larvae (41). Recently, the zebrafish larvae model has been employed to screen 1200 FDA-approved drugs as potential HDTs for TB (42). Mtb-infected lungs in the Wister rat model form well-organized granulomas containing foamy macrophages, epithelioid, and multinucleated giant cells surrounded by a lymphocytic core but this model only occasionally develops caseating lung lesions (43). Mineralization, necrosis, solid caseous centers and hypoxia have been observed in lung lesions of guinea pigs infected with Mtb mimicking human granulomas (44, 45). New Zealand white rabbits develop solid caseous granulomas and liquefied necrotic lesions following Mtb infection (46–48). Goats infected with Mtb develop cavitary lung lesions containing liquefied and solid granulomas but this model is rarely used for anti-TB drug screening (49, 50). The minipig model develops a fibrotic layer that encapsulates lung lesions following Mtb infection and causes intragranulomatous necrosis and calcification of lung lesions entrapping non-replicating mycobacteria within lesions (51). Marmosets infected with clinical isolates of Mtb revealed the full spectrum of lung lesions as observed in TB patients including various degrees of cavitations and extrapulmonary disease (52). Macaque infected with different strains of Mtb, develop the entire spectrum of human TB disease, including active TB, latent infection, chronic progressive infection and the development of spontaneous mineralization of caseous granulomas, as well as fibro-calcific granulomas (35, 53).

*In vitro* Mtb models for human granuloma have been previously developed with peripheral blood mononuclear cells (PBMC) cultured in a collagen matrix (54) or cultured with purified protein derivative of Mtb (55) both forming granuloma-like cell aggregate formation. More recent *in vitro* models of early TB granulomas involve a culture system with tissue-specific epithelial cells and fibroblasts forming structures resembling human lung tissue allowing implantation of Mtb-infected human primary macrophages (56, 57).

In this review, the majority of the listed FDA-approved drugs and inhibitors have been pre-clinically evaluated in mice (19 studies), while 8 HDTs were tested in C3HeB/FeJ Kramnik mice. Despite the limitation of the mouse model such as the lack of caseating granulomas and lung cavities which are the pulmonary pathological hallmarks of human TB, the mouse model still remains instrumental to increase the arsenal of potential HDT candidates that could be clinically evaluated in humans. Currently, only a limited number of HDTs were evaluated in preclinical animal models resembling human granulomas. Among these, we listed three HDTs that were investigated in zebrafish and guinea pigs respectively. Two PDE-4 inhibitors (CC-3052 and CC-11050) and bevacizumab were investigated in rabbits (14, 15, 58) while the IDO inhibitor, 1-methyl-tryptophan and the combination therapy of anakinra and linezolid were studied in macaques (25, 29). Going forward, more HDTs should be evaluated in animal models that mimic human granulomas.

## Molecular Pathways and Specific Targets of HDTs in the Inflammatory Tuberculous Granuloma

### Metalloproteinase Inhibitors

Studies in zebrafish reported that mycobacterial virulence determinants enhance tuberculous granuloma formation (59). More recent studies in zebrafish infected with *Mycobacterium marinum* suggest that granulomas could contribute to the early growth of mycobacteria (60). Here mycobacteria employ the virulence factors expressed from ESX-1/RD1 locus to enhance the recruitment of macrophages to nascent granulomas where they phagocytose infected apoptotic macrophages promoting mycobacterial local expansion and systemic mycobacterial dissemination. In the zebrafish *M. marinum* infection model, the mycobacterial secreted virulence factor, ESAT-6, induces matrix metalloproteinase-9 (MMP-9) in epithelial cells, enhancing the recruitment of macrophages further contributing to nascent granuloma formation and mycobacterial growth (61). Following Mtb infection, MMP-9 deficient mice had decreased mycobacterial burden in the lungs combined with impaired granuloma formation and reduced recruitment of macrophages to the lungs (62). In humans, MMP-9 was highly expressed in tuberculous meningitis (TBM) and pleural TB, both diseases that manifest themselves by extensive tissue destruction (63–65). In addition, MMP-9 inhibitors (Sb-3ct and MMP-9 inhibitor II) increased the mycobacterial killing activity of isoniazid by decreasing the lung burden from Mtb-infected mice (9). Furthermore, mice treated with marimastat, a broad-spectrum MMP inhibitor, enhanced drug delivery and retention of rifampicin plus isoniazid in the lung and decreased blood vessel leakage (9). In addition, marimastat improved the health of blood vessels by increasing pericyte coverage (measured by increased staining of alpha-smooth muscle actin) around the endothelial layer of blood vessels (9). Combination treatment with marimastat and isoniazid synergistically reduced the percentage of consolidated regions within Mtb-infected lung tissue (9). This decrease is most likely due to lower inflammatory immune activation caused by the reduced bacterial load and the activity of marimastat. Thus, the inhibition of MMP activity enabled the normalization of vasculature found in Mtb granulomas which was required to enhance the frontline TB drug delivery to the lungs. Using an experimental TBM infection model, the addition of the specific MMP-9 inhibitor Sb-3ct to anti-tubercular drugs significantly reduced the mycobacterial burden in the brain (66). In a human *in vitro* lung tissue model, inhibition of MMPs by marimastat not only reduced the mycobacterial load but also decreased early granuloma formation characterized by a reduction of the cluster size of monocytes/macrophages at the site of infection (67). The antibiotic doxycycline is currently the only FDA-approved drug to inhibit MMPs for the treatment of periodontitis (68). In a guinea pig model of TB, doxycycline reduced mycobacterial lung burden but did not influence the lung granulomatous involvement (10). In a recent phase II double-blind, randomized, controlled trial, adjunctive doxycycline significantly reduced pulmonary cavity volume, along with decreased MMPs in blood and sputum and reduced the activity of elastase and type 1 collagenase in sputum (69). While most MMP inhibitors have been shown to reduce the pathological lung tissue destruction during TB disease (70), a potent MMP-7 inhibitor cipemastat increased pulmonary cavitation, immunopathology, and mortality in Mtb-infected C3HeB/FeJ Kramnik mice (71). Despite promising preclinical studies, MMP inhibitors failed in phase III clinical trials for cancer, which was mainly attributed to their severe side effects, lack of target specificity, and inadequate knowledge of MMP functions in diseases (72, 73). The development of highly specific third generation MMP inhibitors could potentially alleviate these off-target effects and remain promising drug candidates for cancer and infectious diseases.

### Vascular Endothelial Growth Factor Inhibitors

Mtb infection results in the remodeling of host vasculature to create new blood vessels around granulomas. However, this vasculature of tuberculous granulomas is both structurally and morphologically abnormal with blood vessels displaying heterogeneous spatial densities (58). Vascularization of granulomas provides survival and growth benefits for mycobacteria (74). In the zebrafish *M. marinum* infection model, mycobacteria induce angiogenesis around granulomas promoting mycobacterial growth and dissemination (75). In tuberculous granulomas and cavity walls, hyperactivation of VEGF-A-dependent angiogenesis leads to dysfunctional blood vessel generation, tissue hypoxia, ineffective anti-tubercular drug delivery and inefficient recruitment of immune cells (76). Notably, VEGF production is driven by mycobacteria through the ESX-1 secretion system and cell wall glycolipid trehalose dimycolate (77). This suggests that mycobacteria hijack the formation of new blood vessels for bacterial dissemination (78). Importantly, the pharmacological inhibition of VEGF receptor signaling with pazopanib reduced angiogenesis, mycobacterial burden and limited bacterial dissemination in *M. marinum*-infected zebrafish larvae (75). Mtb-infected human macrophages secrete VEGF and inhibition of VEGF receptor-2 with neutralizing monoclonal antibody in mice strongly reduced the mycobacterial dissemination to the spleen and liver (79). Furthermore, serum levels of VEGF were highly expressed in patients with pulmonary TB (80, 81). Bevacizumab is a full-length recombinant monoclonal IgG anti-VEGF-A antibody that inhibits all VEGF-A isoforms (82). In cancer, bevacizumab is approved by the FDA for the treatment of colorectal, non-epithelial lung, breast, glioblastoma, ovarian and renal cancers (83). In ophthalmology, bevacizumab has been used as an off-label drug. Bevacizumab treatment showed complete resolution of the serous retinal detachment in the eye, which occurs as part of an immune reconstitution inflammatory syndrome (IRIS) in TB-HIV co-infected patients (84). Bevacizumab has the longest half-life (20 hours) compared to other VEGF inhibitors (85). Using bevacizumab to block VEGF created a more structurally and functionally normal granuloma vascularization in the rabbit TB model (58). In this study, bevacizumab increased the blood vessel area per granuloma, enhanced the pericyte coverage and lumen area per blood vessel. In addition, bevacizumab treatment reduced hypoxia in rabbit granulomas whereas the necrotic areas were unaffected. This increased vascular normalization by bevacizumab allowed for enhanced delivery of the Hoechst dye in rabbit granulomas (58). Thus, bevacizumab could potentially increase the delivery of anti-microbial drugs into granulomas. However, bevacizumab treatment did not significantly alter the lung lesion volume, inflammation, or density as measured by FDG uptake, neither was the mycobacterial burden affected (58). VEGF-A regulates excessive granulomatous inflammation and genetic inhibition of VEGF-A improved survival of Mtb-infected mice with concomitant anti-inflammatory effects (76). Pharmaceutical blocking with SU5416, a receptor tyrosine kinase inhibitor of VEGFR1 and VEGFR2, resulted in the reduction of size and density of granulomas with less obstructed alveoli and blood vessels in Kramnik mice. In contrast, granulomas of untreated Kramnik mice were caseating and their hypoxic areas were stained with VEGF-A (76). However, the mycobacterial load was significantly increased in SU5416-treated Kramnik mice when compared to the vehicle control-treated group (76). Anti-VEGF therapy has further shown significant clinical improvement of ocular TB inflammation, suggesting its use in therapy for pulmonary TB (86–88). Angiogenesis is controlled and regulated by multiple host signaling pathways. Targeting uniquely the VEGF/VEGFR-dependent angiogenesis axis might only transiently inhibit angiogenesis due to the upregulation of compensatory signaling pathways as shown in cancer therapy (89). Therefore, the timing and dosing of HDTs are critically important to assess in future clinical trails along with the investigation of other multiple angiogenic pathway inhibitors. Finally, the VEGF-A inhibitor, bevacizumab, is one of the most expensive drugs for cancer therapy and the development of biosimilars will result in a more cost-effective treatment option.

### Antioxidants

Oxidative stress is a common feature in active TB and is related to tissue inflammation. In humans, advanced TB disease was associated with severe oxidative stress with concomitantly reduced antioxidants (90). Oxidative stress progressively increased in lung lesions during Mtb infection of guinea pigs (11). Infected macrophages produce respiratory burst and high levels of reactive oxygen species (ROS) to control and kill Mtb intracellularly (91). However, Mtb has developed survival mechanisms to inactivate ROS responses by producing peroxiredoxin, superoxide dismutase, and catalase enzymes, enabling resistance to ROS of several Mtb strains (92–94). Many first-line anti-tubercular drugs are given to TB patients as inactive prodrugs that are metabolized and activated into pharmacologically active drugs within the host (95). The increase of host oxidants can inactivate these anti-tubercular drugs before they have reached the TB bacilli, effectively neutralizing the antimycobacterial activity (96). Free radicals generated during oxidative burst contribute towards pulmonary inflammation and antioxidants such as N-acetylcysteine (NAC) could potentially reverse these effects. The FDA-approved drug, NAC, is the treatment of choice for paracetamol poisoning (97). It is also prescribed to patients with chronic obstructive pulmonary disease (COPD) or cystic fibrosis (98). As a precursor of the antioxidant glutathione, NAC replenished intracellular levels of glutathione (99). In addition to its known antioxidant properties, NAC reduced mycobacteria growth through various mechanisms including immunomodulation, enhancement of glutathione level, and direct antimycobacterial effects (100). *In vitro* treatment of monocyte-T cell co-cultures with NAC resulted in significantly reduced intracellular growth of Mtb, possibly mediated by NAC immunomodulatory functions on Th1 cytokine secretion (101). Moreover, *ex vivo* treatment with NAC and Mtb infection of whole blood culture isolated from healthy individuals and TB patients resulted in a significant reduction in the intracellular growth of Mtb and pro-inflammatory cytokine secretion (102). Furthermore, NAC treatment of Mtb*-*infected guinea pigs decreased spleen mycobacterial burden, reduced granulomatous lesion and necrosis (11). In the spleen and liver, NAC treatment resulted in granulomas with minimal to no necrosis while untreated guinea pigs contained a large area of necrosis (11). Recently, Mahakalkar et al. clinically evaluated the effect of NAC as an adjunctive therapy during the first two months of standard anti-tubercular therapy in TB patients (103). In this study, adjunctive NAC treatment resulted in early sputum conversion and improved radiological response with the significant clearing of pulmonary infiltrates and cavity size reduction. In addition, glutathione peroxidase levels and body weight were increased in the NAC treatment arm (103).

### Tumor Necrosis Factor Blockers

TNF is an important cytokine in granuloma formation (104). However, high levels of TNF can lead to pathophysiological conditions in the lungs (105). Thalidomide analogues are TNF inhibitors (106) and thalidomide treatment in a placebo-controlled pilot study was well tolerated in patients with pulmonary TB resulting in enhanced body weight gain (107). Adjunctive thalidomide for the treatment of TBM-related complications was safe and clinically effective in children (108). In an experimental rabbit model of TBM, adjunctive thalidomide analog treatment resulted in reduced meningeal inflammation and survival of animals (109, 110). TNF can be further blocked by etanercept, a soluble TNF receptor 2 fusion protein that is widely used in patients for the management of rheumatoid arthritis (111). In C3HeB/FeJ Kramnik mice, adjunctive etanercept reduced necrosis of granulomas and faster resolution of TB lesions when compared to standard TB treatment alone (12). Morphometric analysis showed early decreased lung involvement in Kramnik mice treated with adjunctive etanercept at 4 weeks post-treatment while the early bacterial burden was not changed between the treatment groups indicating that adjunctive etanercept has direct involvement in the early granuloma formation. In the adjunctive etanercept treated group, the bacterial burden only significantly reduced during the continuation phase of TB treatment, killing more slowly multiplying persister TB bacilli when compared to the standard TB treatment group (12). However, TB reactivation has occurred in patients on anti-TNF therapy and therefore, additional studies are required to carefully evaluate the safety of TNF blockers (112, 113).

### Phosphodiesterase Inhibitors

TNF can also be suppressed by PDE inhibitors (114). Cyclic adenosine monophosphate (cAMP) and cyclic guanosine monophosphate (cGMP) are vital second messengers that regulate intracellular signaling pathways which are therapeutically increased by PDE inhibitors for the treatment of various diseases (115). Sildenafil, a type 5 PDE-selective inhibitor, was approved by the FDA for the treatment of erectile dysfunction (116). In a murine TB model, monotherapy with sildenafil or the addition to standard anti-tubercular therapy did not change mycobacterial burden or lung lesions (13). Cilostazol is an FDA-approved PDE-3 inhibitor, antiplatelet drug and a vasodilator that reduces the symptoms of claudication in peripheral artery disease (117). Monotherapy of cilostazol significantly reduced mycobacterial burden in Mtb-infected mice (13). Furthermore, the combination of adjunctive cilostazol and sildenafil decreased the time to lung sterilization by nearly 1 month and decreased pulmonary lesion sizes in mice (13). In murine acute and chronic TB models, the PDE-4 inhibitor roflumilast, an FDA-approved drug for the treatment of COPD exacerbation (118), administered as monotherapy did not affect lung bacillary burden and mortality (119). However, in combination with isoniazid, it reduced mycobacterial burden (119). CC-3052, another PDE-4 inhibitor, in combination with isoniazid (INH) reduced pulmonary pathology and lung CFUs in Mtb-infected rabbits (14). At 8 and 12 weeks post-infection, the number of visible dorsal subpleural lesions was significantly reduced in INH plus CC-3052 treated rabbits. Morphometric analysis of lung tissues revealed that rabbits treated with INH plus CC-3052 had fewer small granulomas, reduced lung lesion numbers and the extent of lung involvement was significantly reduced when compared to the untreated, CC-3052 treated or INH treated group (14). In the INH plus CC-3052 treated group, these small lung lesions were rarely necrotic and included a central core of epithelioid macrophages surrounded by many lymphocytes containing very few acid-fast bacilli and low levels of fibrosis. Although not significant, the number of subpleural lesions, lung lesions and extent of lung involvement was increased and these lung granulomas appeared more cellular, less differentiated and some showed extensive necrosis in the CC-3052 alone treatment group when compared to untreated rabbits (14). Another PDE-4 inhibitor (CC-11050) in combination with isoniazid therapy significantly decreased lung mycobacterial burden and pulmonary pathology in a rabbit TB model (15). INH and CC-11050 treated rabbits had significantly reduced numbers and areas of subpleural lesions with reduced pathology score, while Mtb-infected untreated rabbit lungs exhibited multiple large coalescent lesions with extensive necrosis and some granuloma calcification. Untreated necrotic lesions were surrounded by macrophages, lymphocytes, polymorphonuclear leukocytes, collagen deposition and fibrosis. In contrast, the lungs of INH and CC-11050 treated rabbits contained smaller and fewer granulomas. These lesions had minimal lung fibrosis and absence of acid-fast bacilli and necrosis (15). Subsequently, an open-label phase II randomized controlled trial in patients with pulmonary TB, adjunctive CC-11050 treatment was safe and resulted in improved lung function as measured by the increased recovery of forced vital capacity (FEV_1_) (120). Finally, most of the PDE inhibitors showed efficacy as an adjunctive therapy to standard TB treatment, while many preclinically tested PDE inhibitors in monotherapy did not result in improved disease outcome. This warrants further investigations but also advocates for the use of PDE inhibitors as adjunctive therapy for TB.

### Kinase Inhibitors

Imatinib is a tyrosine kinase inhibitor approved by the FDA for the treatment of chronic myelogenous leukemia (121). Therapeutic or prophylactic treatment with imatinib reduced mycobacterial burden in Mtb-infected mice (122). Imatinib further reduced the bacterial load in mice infected with *M. marinum* and reduced the liver pathology, characterized by a reduction of granulomatous lesions with monocytic infiltrates (122). Further work is required to determine if imatinib directly influences lung granulomatous lesions or if this effect was attributed to a reduced bacterial load. In infected THP-1 macrophages, imatinib in combination with rifampicin synergistically reduced intercellular survival of Mtb (122). CFU counts in the spleen also revealed synergistic antimycobacterial activity of imatinib and rifabutin following *M. marinum* infection (122). Imatinib treatment reduced the pH in lysosomes restricting the growth of Mtb in human macrophages (123). Another tyrosine kinase inhibitor, gefitinib, an epidermal growth factor receptor (EGFR) inhibitor, is used for breast and lung cancers (124). In murine TB models, treatment with gefitinib reduced CFU lung burdens and restricted the intracellular growth of Mtb in macrophages partially by increasing host-protective autophagy (125). Tofacitinib, a Janus kinase inhibitor, is approved by the FDA for the treatment of arthritis in adults and children (126). The addition of tofacitinib to standard anti-tubercular therapy in mice increased time to Mtb clearance by eight weeks as compared to standard treatment alone (127). Ibrutinib is a tyrosine kinase inhibitor used for the treatment of several B-cell malignancies (128). Treatment of mice with ibrutinib significantly reduced Mtb load in mediastinal lymph node and spleen (129). In Mtb-infected macrophages, ibrutinib suppressed the intracellular growth of Mtb by inducing autophagy (129). However, further work is required to investigate the effect of imatinib, gefitinib, tofacitinib, and ibrutinib on pulmonary pathology.

### Histone Deacetylases Inducers or Inhibitors

Sirtuins are histone deacetylase enzymes that play significant roles in post-translational modifications. Natural (resveratrol) and synthetic (SRT1720) sirtuin 1 (SIRT1) activators dampened lung pathology, increased anti-TB drug efficacy and decreased chronic inflammation in Mtb-infected mice (16). Treatment with resveratrol and SRT1720 resulted in smaller granulomatous lesions and reduced involvement of the lung parenchymal area (16). In macrophages, SIRT1 activation decreased the intracellular growth of Mtb, increased phagolysosome fusion and autophagy (16). In contrast, pharmacological inhibition of the histone deacetylase sirtuin 2 with AGK2 in mice, decreased mycobacterial burden, histopathological lung inflammation, enhanced host protective immune against Mtb and increased the efficacy of isoniazid (17). In this study, AGK2 treatment resulted in reduced numbers of quantified granulomas and granuloma pathology scores (17).

### Autophagy Inducers

Autophagy is a host immune mechanism that can suppress inflammation and mycobacterial burden (130, 131). Recently, autophagy-targeting approaches have been suggested as HDT for TB (130, 132, 133). Through a high-throughput screening of a 214 FDA-approved compound library, carbamazepine was identified to induce autophagic killing of intracellular Mtb within human macrophages (18). Juarez et al. confirmed these findings showing that carbamazepine triggered autophagy in both human monocyte-derived macrophages and murine alveolar macrophages (134). Carbamazepine, a sodium channel blocker, is approved by the FDA as an effective treatment for epilepsy, trigeminal neuralgia, and bipolar disorders (135, 136). Importantly, *in vivo* treatment of multidrug-resistant Mtb-infected mice with carbamazepine significantly reduced mycobacterial burden in the lungs and spleen (18). Notably, carbamazepine treatment reduced pulmonary inflammatory infiltrates and decreased lung lesion scores, while mice treated with rifampicin and isoniazid had no effect on pulmonary histopathology (18). However, more work is required to determine if the 2-log decreased Mtb burden could result in the decreased lesion size or if carbamazepine has direct activities on granulomatous lesions.

### Nonsteroidal Anti-Inflammatory Drugs

Ibuprofen and aspirin are NSAIDs that are widely used to relieve symptoms of inflammation, fever and pain (137, 138). Treatment of C3HeB/FeJ Kramnik mice with ibuprofen resulted in decreased number and sizes of lung lesions, reduced the mycobacterial burden, and improved survival (19). Pulmonary histopathology analysis revealed that ibuprofen displayed increased intra-alveolar infiltration of neutrophils and thickened alveolar walls while the control group exhibited extensive central area with caseous necrosis and liquefactive necrosis (19). While ibuprofen treatment alone did not change the mycobacterial burden in mice infected with Mtb (139, 140), the reduced granulomatous lesions in the Kramnik model (19) could be a direct consequence of the anti-inflammatory activity of ibuprofen. Whereas both aspirin and ibuprofen enhanced the antimycobacterial activity of pyrazinamide in mice infected with Mtb (139). However, the combination of isoniazid with aspirin increased CFU counts of Mtb in the spleen and lungs of mice when compared to isoniazid alone (140). In contrast, low-dose aspirin treatment increased mouse survival, reduced pulmonary pathology, and decreased mycobacterial burden in chronic Mtb infection (20). High dose aspirin might be beneficial for the treatment of TBM, hence only 11% of TB patients died or had new brain infarcts as compared to those who received low dose aspirin (15% deaths) or placebo (34% deaths) (141).

### Metformin

Metformin is the first-line medication for type 2 diabetes (142). Mtb-infected mice therapeutically treated with metformin reduced pulmonary tissue pathology and accelerated bacillary clearance (21). Metformin adjunctive therapy with isoniazid potentiated this effect (21). Intracellular growth of Mtb was reduced by metformin-mediated induction of mitochondrial reactive oxygen species and increased phagolysosome fusion (21). In guinea pigs chronically infected with Mtb, metformin treatment decreased lung CFUs and resulted in well-organized granulomas with reduced perilesional inflammation (143). The lung tissue pathology of metformin-treated guinea pigs showed distinct morphological differences in granuloma structure with increased numbers of lymphocytes and reduced peri-lesional spread in the acute and subacute stages of infection when compared to untreated animals (143). Importantly during the acute and subacute infection, the lung Mtb burden did not change between metformin and non-treated groups indicating that metformin had a direct impact on the granulomatous lesions rather than the reduced Mtb growth. Only in the chronic phase of infection, was the mycobacterial burden reduced in the metformin-treated group (143). Systematic reviews evaluated that the prescription of metformin in patients with diabetes mellitus significantly reduced the risk of TB (144, 145). Several clinical trials are currently ongoing to evaluate metformin as an HDT candidate for the treatment of TB (146).

### Vitamins

Administration of calcitriol (the active form of Vitamin D) with pyrazinamide decreased lung bacterial load and attenuated lung lesions in Mtb-infected mice (22). In C3HeB/FeJ Kramnik mice, dietary cholecalciferol decreased the pulmonary immunopathology during the chronic phase of Mtb infection (147). Dietary cholecalciferol further decreased CD3^+^ T lymphocytes staining in lung granulomatous regions during late-stage TB. In contrast, staining of F4/80^+^ (marker of macrophages and myeloid-derived suppressor cells, MDSCs) and Ly6C/Ly6G^+^ (marker of neutrophils and MDSCs) were increased following dietary cholecalciferol in lung granulomas (147). However, several clinical trials revealed that vitamin D supplementation did not shorten the duration of the time to sputum culture conversion (148). All-trans-retinoic acid (ATRA), a metabolite of vitamin A, resulted in reduced mycobacterial load and smaller lung lesion areas in mice or rats infected with Mtb (23, 24, 149). Furthermore, ATRA-treated rats displayed increased numbers of CD4^+^ and CD8^+^ T cells, natural killer cells and CD163^+^ macrophages in the infected lung tissues (24).

### Tryptophan Catabolism and _L_-Isoleucine Amino Acid

In macaques, indoleamine 2,3-dioxygenase (IDO) is greatly increased in the macrophage-rich inner layer of tuberculous granulomas (150). IDO catabolizes the essential amino acid tryptophan into kynurenine degradation products that exert immune suppression such as reduction of IFN-γ production by CD4^+^ T cells (151). The IDO inhibitor, 1-methyl-tryptophan, increased Mtb killing and resulted in the reorganization of the macaque granuloma architecture enabling the trafficking of lymphocytes into the lesion core enabling increased T cell proliferation with increased frequency of granzyme-expressing T cells potentially controlling lung tissue events (25). Furthermore, 1-methyl-tryptophan treatment increased the follicular organization of B cell-containing inducible bronchus-associated lymphoid tissue (iBALT). The lungs of 1-methyl-tryptophan-treated macaques further displayed enhanced T cell function and anti-microbial responses, while untreated animals exhibited T cell exhaustion and dysfunction (25). The administration of the amino acid _L_-isoleucine in mice significantly increased beta-defensins which was associated with reduced lung mycobacterial burden of multi-drug resistant Mtb strains and decreased pulmonary pathology (26). At 4 months post-infection, L-isoleucine treated mice displayed reduced lung consolidation and increased beta-defensins in bronchial epithelium and lung macrophages while untreated mice exhibited extensive pneumonia and very low expression of beta-defensins (26).

### Lactate Metabolism

Lactate dehydrogenase A (LDHA) catalyzes pyruvate into L-lactate during glycolysis. Following Mtb infection, increased levels of both lactate and LDHA have been detected in murine lungs (152, 153), and LDHA was expressed by recruited macrophages within granulomas of C3HeB/FeJ Kramnik mice (154). Administration of LDHA inhibitor (FX11) decreased mycobacterial burden in Mtb-infected mice. Interestingly, FX11 restricted the numbers of necrotic lung lesions, the bacillary burden in Nos2^-/-^ mice and increased the efficacy of isoniazid monotherapy (27). The ameliorated disease pathology following FX11 treatment requires further experimental validation in Kramnik mice that can develop hypoxic necrotic granuloma, because of the confounding effect such as the impaired nitric oxide production in NOS2 deficient mice. The various cell types within granulomas have distinct metabolic activity and this could potentially influence the rate of LDHA inhibition by FX11. Further, FX11 might have potential pleiotropic off-target effects, induce oxidative stress or depriving Mtb energy consumption by inhibiting host-derived lactate (155, 156) which requires further investigations.

### IL-1/Type 1 IFNs Axis

An eicosanoid-based HDT with prostaglandin E2 (PGE2) and zileuton (5-Lipoxygenase inhibitor) treatment in Mtb-infected IL-1α/IL-1β deficient mice resulted in significant decreased mycobacterial loads in bronchoalveolar lavage fluid, diminished lung inflammation, reduced necrotic pulmonary pathology and enhanced survival (28). Furthermore, PGE2 and zileuton treatment did not have any effect in wild-type mice but only in pICLC-treated wild-type mice. In this model, pICLC was used to induce high levels of type I IFNs causing necrotic lung pathology and uncontrolled disease leading to mortality (28). As adjunctive treatment, PGE2 and zileuton did not increase the anti-TB drug efficacy in Kramnik mice (28), thus requiring further preclinical investigations on the potential use of these HDTs for TB. In NOS2 deficient mice, IL-1R1 inhibition with a blocking antibody reduced lung inflammation without changing the mycobacterial lung burden while αIL-1R1 combined with linezolid reduced lung neutrophil numbers in Kramnik mice (29). In macaques, the total lung inflammation measured by FDG activity was significantly reduced following treatment with the FDA-approved soluble IL-1 receptor antagonist anakinra and linezolid when compared to linezolid alone. Furthermore, the combination therapy with anakinra reduced the toxicity of linezolid during Mtb infection (29). The risk of TB reactivation is low during anakinra therapy and so far, only one single case report described the reactivation of previous pulmonary tuberculosis in a patient with rheumatoid arthritis on anakinra (157).

### Myeloid-Derived Suppressor Cells

Lung resident MDSC provide a niche for mycobacterial survival and excessive MDSC accumulation in lungs increases TB lethality in mice (158). The frequency of MDSC is increased in active TB patients suppressing protective T-cell responses (159). Several strategies have been proposed to suppress MDSC which include: pharmacological targeting, reversing MDSC impact on anti-TB immunity, inhibition of MDSC expansion/recruitment, targeting functions and changing the maturation/differentiation of MDSC into non-suppressive cells (160). Although etanercept, anti-VEGF, tyrosine kinase inhibitors, PDE-5 inhibitors among others have been experimentally validated in cancer to suppress MDSC, more experimental Mtb studies are required to determine the impact of these HDTs on MDSC functionality (160).

### Statins

Statins with over 100 million prescriptions worldwide, lower cholesterol in cardiovascular disease (161). Statins inhibit HMG-CoA reductase, the rate-controlling enzyme of the mevalonate pathway (162). In addition, statins also have broad-range immune-modulatory and anti-inflammatory properties with potential use as HDT against infectious diseases (163). We previously reported in mice that statins reduced Mtb burden by enhancing autophagy, phagosome maturation and decreasing pulmonary pathology with fewer and smaller lesion sizes (30), suggesting a role for statins as HDT in TB (164). Others reported that statins as adjunctive therapy reduced the time to TB cure and decreased mouse lung pathology with smaller lung lesions (165, 166). In C3HeB/FeJ Kramnik mice, adjunctive statins decreased lung CFUs and the percentage of lung surface area involved by inflammation (31). Several population-based studies reported that statin treatment was associated with a decreased risk of TB disease (167–170). Pravastatin is currently being evaluated in a clinical phase 2b dose-finding study in adults with TB (StAT-TB, ClinicalTrials.gov identifier: NCT03882177). In a phase IIB, double-blind, randomized, placebo-controlled trial we are currently testing atorvastatin to reduce inflammation after TB treatment completion (StatinTB, ClinicalTrials.gov identifier: NCT04147286).

### Others

A recent study screened 1200 FDA-approved drugs as potential HDTs in zebrafish larvae infected with *M. marinum* (42). Using an *ex vivo* granuloma explant model, this study identified clemastine, an antihistamine drug, to reduce the mycobacterial growth within complex established granulomas through potentiation of the purinergic receptor P2RX7 (42). However, clemastine did not influence the granuloma structure (42). Many other immunomodulatory HDTs for TB have been shown to target host lipid glycolysis and lipid metabolism in macrophages, however, further investigations in animals are required to assess their activity in reducing TB granulomatous lung lesions (171).

## Potential Limitations of HDTs

Prospective drawbacks of HDTs for TB include potential off-target effects, drug-drug interactions, and associated side effects. These limitations require further investigations in future preclinical and clinical studies. TB is a complex disease that still needs to be fully characterized, therefore the impact of HDTs on TB necessitates further detailed evaluations. As TB progresses from quiescent infection to incipient, a subclinical and active disease, many HDT host targets are differentially expressed during this spectrum. More specifically, in the lungs, during the formation of granuloma, HDT targets can be disparately expressed. Many preclinical studies have only investigated a few time points in the expression kinetics of HDT targets during Mtb infection. Therefore, the dosing and timing when host-directed therapy could start during infection should be investigated more in detail. Furthermore, the heterogenous lung lesions that coexist in the same individual remain challenging for effective HDT treatment. In patients, HDTs should be administered as adjunctive with standard TB therapy and many TB patients are also co-infected with HIV. This remains challenging because anti-TB drugs such as rifampicin induce cytochrome P450 enzymes. Many antiretroviral drugs can either induce or inhibit cytochrome P450 enzymes which can differentially metabolize HDTs leading to increased or decreased drug bioavailability and induce potential adverse events such as drug toxicity. Therefore, during designing clinical trials, consultation of guidelines such as European AIDS Clinical Society guidelines and clinical pharmacokinetic studies are critically important to assess in TB/HIV co-infected patients. Finally, the effective treatment outcome of HDTs could differ among TB patients because of the genetic composition of individuals as demonstrated in studies where polymorphisms were linked to resistance or susceptibility to Mtb infection. As a precision medicine approach, identification of host prognostic biomarkers could potentially identify TB patients that are most likely to respond positively to the treatment outcome of HDTs.

## Conclusion and Future Perspective

Post-TB lung damage persists despite microbiological cure in pulmonary TB caused by post-TB destructive lung disease due to chronic fibro-cavitation, bronchiectasis, COPD, lung collapse, and pulmonary hypertension (172–174). Persistent pulmonary inflammation and ongoing paucibacillary Mtb replication have moved into the focus of adjunctive HDT research (175–181). This current review extends the body of literature with a particular focus on HDT candidates that reduce lung granulomatous lesions and pulmonary inflammation. FDA-approved drugs for human use can be repurposed as adjunctive HDT for TB and accelerate the clinical development of novel TB treatment regimens. Ultimately, randomized-controlled trials are required to test HDT candidates in the context of TB to evaluate the safety and potential drug-drug interactions, dosing and treatment duration, and treatment outcome. If successfully employed, effective HDT candidates may lead to TB treatment shortening and improved quality of life in patients with TB by preventing lung destruction and ameliorating lung functions. Hence, HDTs may enhance TB treatment and contribute to the ambitious target of the World Health Organization to end the global TB epidemic by the year 2035.

## Author Contributions

RG wrote the review with substantial, direct, and intellectual contribution from all authors. RG created **Figure 1**. SS, BM and RG created **Table 1**. All authors contributed to the article and approved the submitted version

## Funding

This publication was produced by StatinTB which is part of the EDCTP2 programme supported by the European Union (grant number RIA2017T-2004-StatinTB) to RG and FT. Research reported in this publication was supported by the National Institute Of Allergy And Infectious Diseases of the National Institutes of Health under Award Number R01AI160501 to RG. This work was supported by the grants from NRF/DST-South African Research Chair Initiative (SARCHi), South African Medical Research Council (SAMRC) and International Centre for Genetic Engineering and Biotechnology (ICGEB) awarded to FB. The work was supported by the Wellcome Trust CIDRI-Africa 203135Z/16/Z fund.

## Author Disclaimer

The views and opinions of authors expressed herein do not necessarily state or reflect those of EDCTP and Wellcome Trust. The content is solely the responsibility of the authors and does not necessarily represent the official views of the National Institutes of Health.

## Conflict of Interest

The authors declare that the research was conducted in the absence of any commercial or financial relationships that could be construed as a potential conflict of interest.

## Publisher’s Note

All claims expressed in this article are solely those of the authors and do not necessarily represent those of their affiliated organizations, or those of the publisher, the editors and the reviewers. Any product that may be evaluated in this article, or claim that may be made by its manufacturer, is not guaranteed or endorsed by the publisher.
